# Application of the GARC Data Logger—a custom-developed data collection device—to capture and monitor mass dog vaccination campaigns in Namibia

**DOI:** 10.1371/journal.pntd.0008948

**Published:** 2020-12-28

**Authors:** Rauna Athingo, Tenzin Tenzin, Andre Coetzer, Emmanuel H. Hikufe, Josephat Peter, Laina Hango, Tangeni Haimbodi, Johannes Lipinge, Frenada Haufiku, Matias Naunyango, Magano Kephas, Albertina Shilongo, Kenneth K. Shoombe, Siegfried Khaiseb, Moetapele Letshwenyo, Patricia Pozzetti, Lorenz Nake, Louis H. Nel, Conrad M. Freuling, Thomas Müller, Gregorio Torres

**Affiliations:** 1 Animal Disease Control, Sub-division, North-West, Directorate of Veterinary Services (DVS), Ministry of Agriculture, Water and Land Reform, Ongwediva, Namibia; 2 World Organisation for Animal Health (OIE), Sub-Regional Representation for Southern Africa, Gaborone, Botswana; 3 Global Alliance for Rabies Control (GARC), Pretoria, South Africa; 4 Department of Biochemistry, Genetics and Microbiology, Faculty of Natural and Agricultural Sciences, University of Pretoria, South Africa; 5 Directorate of Veterinary Services (DVS), Ministry of Agriculture, Water and Land Reform, Windhoek, Namibia; 6 Outapi State Veterinary Office, Directorate of Veterinary Services (DVS), Ministry of Agriculture, Water and Land Reform, Omusati region, Outapi, Namibia; 7 Ondangwa State Veterinary Office, Directorate of Veterinary Services (DVS), Ministry of Agriculture, Water and Land Reform, Oshana region, Ondangwa, Namibia; 8 Omuthiya State Veterinary Office, Directorate of Veterinary Services (DVS), Ministry of Agriculture, Water and Land Reform, Oshikoto region, Omuthiya, Namibia; 9 Eenhana State Veterinary Office, Directorate of Veterinary Services (DVS), Ministry of Agriculture, Water and Land Reform, Ohangwena region, Eenhana, Namibia; 10 Central Veterinary Laboratory, Directorate of Veterinary Services (DVS), Ministry of Agriculture Water and Land Reform, Windhoek, Namibia; 11 World Organisation for Animal Health (OIE), Paris, France; 12 Institute of Molecular Virology and Cell Biology, Friedrich-Loeffler-Institute, Greifswald—Insel Riems, Germany; Environment and Sustainability Institute, UNITED KINGDOM

## Abstract

Domestic dogs are responsible for 99% of all cases of human rabies and thus, mass dog vaccination has been demonstrated to be the most effective approach towards the elimination of dog-mediated human rabies. Namibia demonstrated the feasibility of this approach by applying government-led strategic rabies vaccination campaigns to reduce both human and dog rabies incidences in the Northern Communal Areas of Namibia since 2016. The lessons learnt using paper-based form for data capturing and management of mass dog vaccination campaign during the pilot and roll out phase of the project (2016–2018) led to the implementation of a simple and accurate data collection tool in the second phase (2019–2022) of the rabies elimination program. In this paper, we describe the implementation of such custom-developed vaccination tracking device, i.e. the Global Alliance for Rabies Control (GARC) Data Logger (GDL), and the integration of the collected data into a website-based rabies surveillance system (Rabies Epidemiological Bulletin—REB) during 2019 and 2020 campaigns. A total of 10,037 dogs and 520 cats were vaccinated during the 2019 campaign and 13,219 dogs and 1,044 cats during the 2020 campaign. The vaccination data were recorded with the GDL and visualized via REB. Subsequent GIS-analysis using gridded population data revealed a suboptimal vaccination coverage in the great majority of grid cells (82%) with a vaccination coverage below 50%. Spatial regression analysis identified the number of schools, estimated human density, and adult dog population were associated with the vaccination performance. However, there was an inverse correlation to human densities. Nonetheless, the use of the GDL improved data capturing and monitoring capacity of the campaign, enabling the Namibian government to improve strategies for the vaccination of at-risk areas towards achieving adequate vaccination coverage which would effectively break the transmission of rabies.

## Author summary

We used a custom-developed vaccination tracking device—the Global Alliance for Rabies Control (GARC) Data Logger—to capture dog rabies vaccination data during the 2019 and 2020 mass vaccination campaign in the Northern Communal Areas of Namibia, and then integrated the collected data into the web-based Rabies Epidemiological Bulletin, a rabies-specific disease surveillance platform for rabies-endemic countries. This approach allowed automatic collation, analysis and, visualization of data and drastically improved the data capturing and monitoring capacity of the Namibian government led campaign. Additionally, subsequent GIS analysis enabled a better estimation of vaccination coverage at a much higher spatial resolution, thus identifying areas where improvements in the vaccination strategy are needed to ensure long-term success of the project.

## Introduction

Rabies, caused by viruses of the Lyssavirus genus, of which rabies lyssavirus (RABV) is the prototype species [1,2], has the highest case fatality rate of any known infectious disease. Rabies is also one of the deadliest diseases responsible for around 59,000 human deaths each year; with over 95% of the cases contracted from a dog bite [3]. While dog-mediated rabies is endemic to many developing countries globally, the highest rabies burden is in Africa and Asia which accounts for 95% of rabies deaths worldwide [3–5].

The elimination of dog-mediated human rabies is integral to the United Against Rabies (UAR) collaboration, which involves four partners: the World Health Organization (WHO), the Food and Agriculture Organization of the United Nations (FAO), the World Organisation for Animal Health (OIE) and the Global Alliance for Rabies Control (GARC), which work at the animal–human–systems interface [4,6,7]. The UAR recognized that a lack of operational research has led to knowledge gaps in how to design and implement control and elimination programs where they are needed most [8–10]. To move from the biological understanding of the disease to operational science and policy [11] the UAR, together with other international partners, proceeded to take a leading role in the development and deployment of strategies needed to eliminate rabies as a cause of human suffering and death as part of the Zero by 30 initiative [12].

One example of a country that has implemented a large-scale government-driven rabies control programme is Namibia. Rabies has been endemic to Namibia since at least 1887, with the first case being diagnostically confirmed in 1906 [13,14]. Both dog-mediated and sylvatic rabies is endemic in Namibia. While sylvatic rabies is found throughout Namibia, dog-mediated rabies is restricted to the eight Northern Communal Areas (NCAs), which accommodate approximately 60% of the human population in Namibia [15].

**Fig 1:**
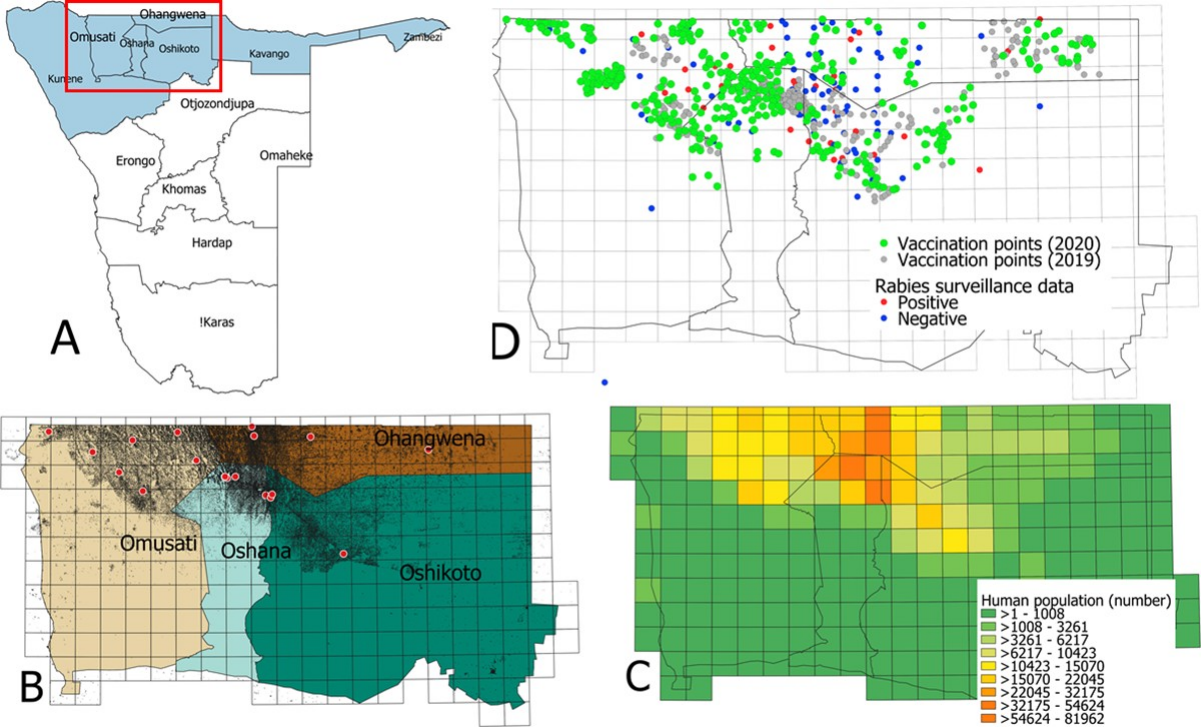
Study area for mass dog vaccination campaigns in Namibia. Map depicting the location of the greater study area (red rectangular) in Namibia and the boundaries and names of the Namibian regions (**A**), grid cells (20 x 20 km) based map of the enlarged area showing the human population distribution (black dots) in the four study regions with the location of towns (red dots) and the rest of the areas are the rural settlements (**B**), grid level human population number (**C**), and the cumulative rabies surveillance data for the time period 2018–December 2019 and the exact location of vaccination points during 2019 and 2020 vaccination campaigns (**D**).

Similar to other African countries, i.e. Uganda [16], Republic of South Africa [17], Tanzania [18], Malawi [19], and Kenya [20], the Namibian government launched a National Rabies Control Strategy in March 2015 [21]. The resulting dog rabies elimination programme targeting the eight regions of the Northern Communal Areas (NCAs, Oshana, Oshikoto, Omusati, Ohangwena, Kunene, Kavango West, Kavango East and Zambezi) (Fig 1A) has been co-financed by the German government and receives technical support from the OIE and the Friedrich-Loeffler-Institut (FLI), Germany. To this end, mass dog vaccination campaigns were started in a pilot project area, *viz*. the Oshana region in 2016 [21]. The vaccination coverage in dogs and decreasing trends in disease prevalence achieved during the pilot project [15,21], prompted the stakeholders to roll out the mass dog vaccination campaigns to the remaining NCAs in a stepwise manner from 2017 onwards [21].

More specifically, the mass dog vaccination campaign was rolled out in all eight regions of the NCA (Oshana, Oshikoto, Omusati, Ohangwena, Kunene, Kavango West, Kavango East and Zambezi) during 2017 and 2018. During this time, a total of 99,814 dogs and 10,538 cats were vaccinated in 2017 and 72,953 dogs and 8,710 cats in 2018, achieving an estimated coverage of 73.1% across the NCA region [21]. As a result of the first phase of the National Rabies Control Strategy across the eight region of the NCA–an area that was once highly endemic for rabies–rabies cases in dogs and humans had decreased [15,21]. Indeed, the proportion of rabies positive samples has been significantly reduced over the years and the rabies cases in dogs had decreased from over 90 cases between 2015 and 2016 to 34 in 2018. A similar trend was observed in the incidence of human rabies cases, with the number of confirmed human rabies deaths decreasing from 23 cases in 2015 to 6, 4, and 1 in 2016, 2017 and 2018 respectively [15,21].

Despite the promising results and the encouraging initial success, key lessons were learned and areas of improvement were identified as experienced elsewhere in African and Asian rabies control programs [18,22,23]. One challenge identified particularly during the roll out phase of the program was the collection of dog vaccination and other related data mainly on standard paper-based forms [21]. To overcome limitations in efficient and timely collection of dog rabies vaccination data and subsequent analysis, we initiated the implementation of a portable custom-developed data-capturing device–the GARC Data Logger (GDL) and the integration of captured data into the website-based Rabies Epidemiological Bulletin (REB) platform [24,25] to improve monitoring and optimization of future mass dog vaccination campaigns (MDV). Here, we describe the experiences on the application of data logger for the near real time management of MDV data during 2019 and 2020 campaign in Namibia. In furtherance, we also aim to showcase how dog vaccination data collected with the GDL, combined with the human population density maps, could be used to determine a localized estimated dog vaccination coverage–an approach that has, to the best of our knowledge, not been implemented to date.

## Methods

### Ethics statement

The mass dog vaccination campaign was conducted as part of a non-research public health intervention to eliminate dog-mediated rabies in Namibia since 2016. Therefore, ethics approval is not required for the analyses of the campaign data.

### Study area

The use of the new data collection tool was exemplarily implemented in four neighboring regions of the NCAs including Oshana, Oshikoto, Omusati and Ohangwena (Fig 1A), in which targeted MDV campaigns were conducted in selected rabies hot spot areas during August/September 2019 and June/July 2020. The study area comprising of 22,000 km^2^ was set up in regions of good access with high human density and high numbers of reported rabies cases based on a 20 km X 20 km grid cell basis covering both urban and rural settings (Fig 1B and 1C). The 2018–2019 rabies surveillance data (laboratory diagnoses cases) maintained in the national animal disease database were used to map and identify the rabies hotspots for targeting mass dog vaccination campaign (Fig 1D).

### GARC Data Logger (GDL) training and application in Namibia

Twenty GDL devices were procured to capture and monitor the MDV activities. The GDL was developed as a labile, hand-held data collection device that consist of a robust hard-wearing plastic casing, touch-activated buttons and indicator light-emitting diodes (LEDs), which make it rather resistant to damage and breakage [24]. The GDL can collect the most important data associated with every vaccinated animal (animal species, sex and age) in the field within approximately three seconds. Once data capturing has been confirmed, the GDL also automatically records the GPS coordinates using satellite triangulation, as well as the date and time of vaccination. Data that was captured and stored on the device (with an internal memory that can store up to a maximum of 500 recordings) is subsequently connected to a computer using a universal serial bus (USB) type-C cable and downloaded using a custom-developed software program called the “GDL Manager” (version 1.11) [24].

Prior to the implementation of the GDL devices, a one-day training for the vaccination teams on the GDL application and data management was provided in the four NCA regions at their respective State Veterinary Offices. All of the relevant staff were trained on downloading and transferring the data to the Rabies Epidemiological Bulletin (REB). The REB is a free-to-use web-based data platform that was developed by GARC in 2016 using the District Health Information System 2 (DHIS2) platform [24,25]. The main purpose of the REB is to provide governments of rabies-endemic countries with a data analysis platform that facilitates the establishment and routine use of rabies-specific data–allowing the governments to make data-driven decisions with regards to their own rabies elimination efforts [25]. In addition to managing the national-level data, the REB also has additional functionality that allows governments to effectively manage data that has been collected during dog vaccination campaigns, human rabies treatment events and active or passive rabies surveillance campaigns. For example, the REB enables data that has been collected during dog vaccination campaigns to be automatically displayed as vaccination points on various interactive maps as shown in S1 Fig, while also automatically generating graphs of the total number of vaccinated animals with the data disaggregated by species, age and sex. By utilizing the REB’s functionality the campaign managers and the veterinary authorities that had been granted the relevant permission could visualize the vaccination data in near real time. This allowed the relevant authorities to monitor where vaccination campaign is being implemented, while also being able to share the data with any other stakeholders.

### Operational implementation of the mass dog vaccination campaigns

The state veterinarians and animal health technicians of Directorate of Veterinary Services (DVS) of the NCA regions conducted the vaccination campaign as described previously [21]. Briefly, several strategic vaccination points were prepared at 1–3 km intervals within each of the identified communities of the Oshana, Omusati, Ohangwena and Oshikoto regions, focusing on rabies hotspots areas where outbreaks were known to occur (Fig 1D). A total of 13 vaccination teams (consisting of two members per team) conducted the campaign from 12 August to 11 September (22 working days) in 2019, and the campaign ran from 20 June to 24 July in 2020 (16 working days, 17 vaccination teams) in the above four regions. The vaccination campaigns were conducted during school holidays in 2019 and during the closure of schools in 2020 because of the SARS-CoV-2 pandemic. This ensured that the students were available for bringing dogs to the vaccination centres during both campaigns. For this purpose, high-quality inactivated vaccines manufactured according to OIE international standards were procured through the OIE Vaccine Bank. Vaccine cold chain was maintained using cold boxes (2–8°C) during transport to the vaccination centres. The government of Namibia funded the logistics cost of the campaign which included vaccination team transports, per diem payment, syringes, needles, gloves, and vaccination certificates. Funds donated by the German Federal Ministry of Food and Agriculture were managed by OIE to procure rabies vaccine, GDL devices, training, and communication materials. The vaccination campaigns were announced in advance by posters and radio broadcasts and using loudspeaker announcements on the day of vaccination at the vaccination centres. The vaccination of dogs was provided free of charge and the GDL devices were used to record the vaccination data, which was captured by species (dog vs cats), age (adult vs juvenile) and sex (male vs female). Those dogs approximated to under one year old were recorded as juvenile and above one year as adults. The campaign manager from the respective state veterinary offices, including the rabies project coordinator monitored the vaccination campaign remotely by login into the REB using unique login credentials.

### Vaccination coverage estimation

At the time of writing, no dog census had been conducted in Namibia and the dog population was thus not well-defined–complicating the estimated vaccination coverage. Post-vaccination surveys were also not conducted due to logistical and financial constraints. In order to estimate the dog vaccination coverage using a novel approach, a gridded population density of Namibia estimated for the year 2020 was obtained from the Center for International Earth Science Information Network (CIESIN 2018) as a raster file containing the number of people per 1 km grid square (Fig 1B). Subsequently, a 20 by 20 km grid was laid over the NCA region map and the human population in each grid cell of the study area was calculated (Fig 1B). Following on from this, the dog population per grid cell was estimated according to the assumed human:dog ratio of 8.3:1 [21]. Data on the number of individual dogs vaccinated in the four NCA regions as captured by the GDLs was also linked to the same grid cells and the estimated vaccination coverage was calculated per grid cell.

The bar graph, box plot and 95% binomial confidence interval (CI) for each grid cell vaccination coverage was calculated and plotted as error bars in R version 3.6.1 [26]. The data from three grid cells in 2019 were excluded from the analysis since they contained outliers likely due to inaccuracy in the estimated dog population (see regression analysis below). The vaccination data aggregated by age and sex were also analysed. The vaccination team efficiency was also assessed by calculating the daily/hourly number of animals vaccinated by each team. All spatial analyses including extraction of the human population and vaccination data from each grid cell, vaccination coverage estimation and mapping were performed using the count by polygon vector analysis feature in Quantum GIS version 3.8.3 (QGIS Development Team 2019, Open Source Geospatial Foundation Project, http://qgis.osgeo.org).

### Regression analysis of vaccination coverage

Regression analysis was performed to determine the predictors associated with the outcome variable. In our analyses, the outcome variable was the estimated vaccination coverage achieved in each of the 20 x 20 km grid cells calculated as the number of dogs vaccinated divided by the estimated dog population as described above. The predictor variables that were considered were the estimated human population, estimated dog population, number of schools (S2 Fig), number of rabies positive cases in dogs during 2018–2019, and the number of dogs (juvenile, adult, male, female dogs) presented to the vaccination centres per grid cell. Each predictor variables were calculated and extracted using the count by polygon vector analysis feature in Q GIS, as described above. Because the vaccination campaign was conducted by vaccination posts with varying vaccination points in different grid cells, we assumed that in some instances the dog owners from outside the cells could have brought dogs to another nearby cell for vaccination. Thus, the number of dogs vaccinated per cell would not have necessarily corresponded with the number of vaccinated dogs residing in the cell. In addition, because of high variability in the estimated dog population (denominator data), the grid cell vaccination coverage rates were smoothed using a spatial rate in a moving window centered on each grid cell in turn. The moving window included the grid cell as well as its neighbors thereby taking into account the vaccination coverage observed at neighboring cell. The neighbors were defined using the rook continuity spatial weights, which was created based on the grid cell sharing border with the neighboring cells. All analyses were conducted using GeoDa 0.9.5-i5 (Beta) (https://geodacenter.github.io/) [27]. The details of the spatial regression analyses and model diagnostics used to select the best fit model are described in S1 Appendix.

## Results

### Mass dog vaccination campaign and data capturing using GDL device

In the study area, during 22 working days in August/September 2019 vaccination campaign, a total of 10,037 dogs and 520 cats were vaccinated by 13 vaccination teams (two members per team) at 167 temporary vaccination centres. This equates to an average vaccination efficiency of 480 animals per day (37 animals per day per team and five animals per hour per team). The daily vaccination campaign ran from around 6.00AM to 4.00PM but 90% of the vaccination was delivered between 7.00AM and 1.00PM (S3 Fig). Of the total number of dogs vaccinated, 55% (5,506) were males and 45% (4,531) females of which 53% (5,280) were adult dogs and 47% (4,757) were juveniles (Table 1). During the June/July 2020 vaccination campaign (16 working days), 17 vaccination teams (two member per team) covered 486 vaccination points and vaccinated 13,219 dogs and 1,044 cats—equating to an average vaccination efficiency of 891 animals per day (53 animals per day per team and six animals per hour per team). Similarly, the daily vaccination campaign ran from 6.00AM to 4.00PM and 94% of the animals were vaccinated between 7.00AM and 1.00PM (S3 Fig). The time distribution of animals vaccinated at the vaccination centres is shown in S3 Fig. Of the total dogs vaccinated, 58% (7,691) were males and 42% (5,528) females of which 65% (8,616) were adult dogs and 35% (4,603) were juveniles (Table 1).

**Table 1:** Total number of dogs vaccinated aggregated by age and sex during 2019 and 2020 vaccination campaign in the four NCA regions of Namibia.

Age	2019 campaign		Total (%)	2020 campaign		Total (%)
	Female (%)	Male (%)		Female (%)	Male (%)	
Adult (>1 year)	2,245 (42.5)	3,035 (57.5)	5,280 (52.6)	3,412 (39.6)	5,204 (60.4)	8,616 (65.2)
Juvenile (<1 year)	2,286 (48.1)	2,471 (51.9)	4,757 (47.4)	2,116 (45.9)	2,487 (54.1)	4,603 (34.8)
**Total**	**4,531 (45.1)**	**5,506 (54.9)**	**10,037**	**5,528 (41.8)**	**7,691(58.2)**	**13,219**

**Fig 2:**
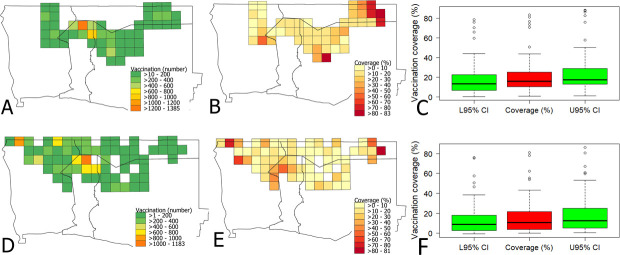
Maps and box plots showing vaccination and vaccination coverage. Grid cells (20 x 20 km) based map of the study area showing the number of dog vaccinated (**A**), vaccination coverage achieved per grid cell (**B**) and boxplot of the overall vaccination coverage and 95% confidence interval during the 2019 MDV campaign (**C**). The 2020 vaccination campaign data is represented by the number of dogs vaccinated (**D**), vaccination coverage achieved per grid cell (**E**), and boxplot of the average vaccination coverage and 95% confidence interval (**F**).

**Fig 3:**
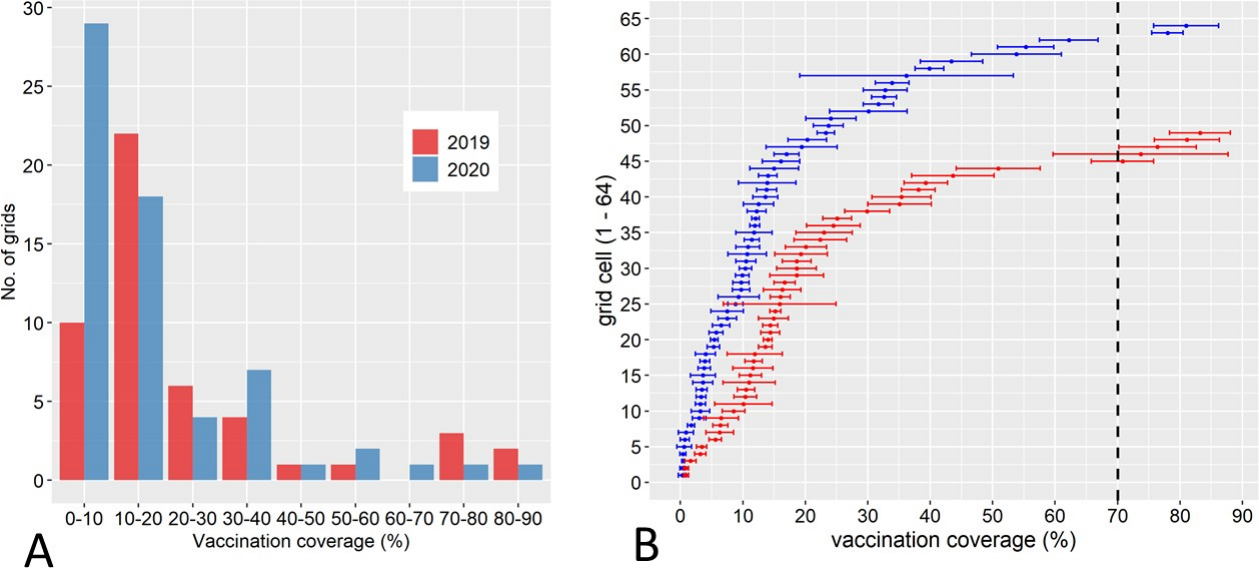
Bar graph and plots of vaccination coverage by grid cells. Correlation between number of grid cells and dog vaccination achieved during the 2019 and 2020 MDV campaign in the four regions of NCA, Namibia (**A**). A plot of dog vaccination coverage (%) per grid cells with 95% confidence intervals presented as horizontal bars for 2019 (red) and 2020 (blue) vaccination campaign is shown in (**B**). The vertical dashed line represents the 70% recommended threshold.

The mean population density per km^2^ for the 2019 study area amounted to 24 people on average, ranging from 0.2 to 204 and 30 people (range: 0.6 to 204) for the 2020 campaign area. When the estimated human:dog ratio of 8.3:1 was applied, the overall vaccination coverage achieved during the 2019 campaign was 23.01% (95%CI: 16.90–29.25) (Fig 2A–2C) with the great majority of the grid cells (87%) containing a suboptimal vaccination coverage (<50%) during 2019 campaign (Fig 3). Similarly, during the 2020 campaign, only 16.64% (95%CI: 12.09–21.18) coverage was achieved (Fig 2D–2F) with 92% of the grid cells containing a vaccination coverage below 50% (Fig 3). The 2020 campaign, however, covered 64 grid area compared to 49 grids during 2019 (Fig 3). Only few grid cells area (*n = 7*) have achieved more than 70% coverage threshold required to break the transmission chain among dogs (Fig 3).

### Regression results

The details of an OLS regression model and the spatial (Lag and Error) models are presented in S1 and S2 Tables. Comparing to the OLS and the spatial error model, the spatial lag model emerged as the best fit model (Table 2) that explain the vaccination coverage rate. The spatial lag model containing the number of schools, estimated human population, and the number of adult dogs fit the vaccination coverage performance during 2020 campaign while the estimated human population and the number of adult dogs fit the 2019 vaccination coverage performance (Tables 2, S1 and S2). In our investigation, there was an inverse relationship between the population density and vaccination coverage, indirectly suggesting that there would be a low coverage with increasing dog population if the existing human:dog ratio is considered. However, the model residuals (heteroskedasticity) and spatial lag dependence were still significant, which indicates that the spatial effects in the data have still not been removed completely. This suggest that a further consideration is needed of alternative model specifications, either including new explanatory variables or incorporating different spatial weights, but we did not incorporate the new explanatory variables in our analysis due to lack of data. The observed and predicted smoothed log-transformed grid level vaccination coverage rates were highly correlated (r:0.8995, P<0.0001) for 2019 data and moderately correlated for 2020 data (r:0.5651, P<0.0001) indicating the fit of the spatial lag model. A detailed explanation on regression diagnostics and model selection including the results of the models is presented in the S1 Appendix.

**Table 2:** The variables associated with a spatial lag model of log-transformed vaccination coverage against rabies in dogs in NCA regions, Namibia.

*Variable*	*Coefficient*	*SE*	*Z-value*	*P-value*
***2020 vaccination data***				
Spatial Lag (Rho)	0.39303	0.11049	3.557	0.00037
Constant	0.75294	0.39858	1.88905	0.05888
Number of schools	0.31687	0.15336	2.06613	0.03881
Human population	-0.47104	0.13865	-3.39733	0.00068
Adult dog population	0.15184	0.04261	3.56349	0.00037
***2019 vaccination data***				
Spatial Lag (Rho)	0.53777	0.10826	4.96702	0.00007
Constant	0.25664	0.15208	1.68758	0.09149
Human population	-0.26109	0.05926	-4.40546	0.00001
Adult dog population	0.21522	0.05176	4.15735	0.00003

## Discussion

The use of various applications of portable handheld computer technology and other sophisticated data collection tools including Open Source software Epi Info [28], EpiCollect [29,30] and mobile phone technology [31–35] in the field of veterinary and public health has been replacing traditional paper and pencil modes of data capture. These data collection tools and devices are increasingly used, particularly in developing countries where the technology is becoming increasingly available. Mobile phone applications even allow for real time monitoring of vaccination campaigns performance [31,32]. For rabies surveillance and monitoring of MDV campaigns, special tools have been developed [24,32–35]. Although this so-called handheld technology has several tangible advantages over traditional paper and pencil modes of data capture including, but not limited to: data accuracy; timeliness of data capture; and adherence to protocols for data collection [36], its use in the Namibian dog rabies elimination program was initially not considered necessary. However, the program faced challenges insofar that data were partly inconsistent and had to be processed manually into a software exploitable format. This prevented timely sophisticated data analyses and the identification of poorly performing areas that would require repeated vaccinations.

Hence, to meet future demands on data processing and analysis in the frame of upcoming large-scale MDV campaigns, the introduction of automated data capturing was inevitable. Our study demonstrates that the introduction and use of the GDL and subsequent integration of captured data into a central data repository, e.g. the REB, was similarly successful as experienced in the Zanzibar Archipelago and the Harare Province of Zimbabwe [24,25]. The automated data collection and the link to the individual geo-coordinates for the first time allowed a standardized and timely display of data via the REB as shown in S1 Fig [25].

When processed using GIS-tools, the data could be even more informative, especially when linked to other denominator data. While previous dog vaccination coverage estimates were based on local or regional administrative units [21], the exact location of every dog vaccination with the estimated human density allowed for a vaccination coverage estimation at a much higher spatial resolution (20x20 km). Thus, spatial clustering of gaps in vaccination which may be camouflaged when data is accumulated, becomes more evident and mitigating measures can be precisely targeted.

The MDV campaign of 2019 was constrained by logistical and financial issues, restricting the vaccination to regions of good access and high numbers of reported rabies cases (Fig 1D). Similarly, the 2020 campaign had to be conducted under difficult circumstances due to SARS-CoV-2 pandemic. Since the vaccination teams and the pet owners were required to follow social distancing and public gathering was not allowed, the execution of the vaccination programme was considerably more challenging than would otherwise have been the norm. Despite these limitations, high numbers of dogs were vaccinated during both the MDV campaigns in the study areas. The results also demonstrated maximum vaccination between 7.00AM and 1.00PM suggesting that future vaccination should be planned during these time period. One essential component for estimating the vaccination coverage is the actual number of dogs as the denominator. We used an estimated human:dog ratio of 8.3:1 from previous assessments in the vaccination area [21]. Interestingly, this ratio is very close to data from rural Tanzania [37], presumably because of similar socio-cultural settings. Also, these ratios are within the range of other human:dog ratios documented for Africa [38–40]. Using this ratio for the dog population estimate, the vaccination coverage in large parts of the study area was still suboptimal or even inadequate (Figs 2 and 3), considering that a vaccination coverage of at least 70% amongst at-risk dog populations is needed to achieve control and eventual elimination of rabies in a given area [41,42]. Also, the high percentage of juvenile dogs indicated a high population turn-over which further complicated elimination efforts, as the proportion of susceptible animals remained high.

Understanding the factors that determine the success/failure of vaccination campaigns and the achieved vaccination coverage plays a vital role in planning and supporting effective rabies control programme [19,43]. As such, we utilized regression modelling to provide insights into which predictor variables were related to the campaign performance in Namibia. Our regression models identified three specific predictors that were associated with the vaccination coverage achieved at the grid cells level, *viz*. the number of schools, human population density and the adult dog population. In our investigation, the impact of the school placement could be explained by the fact that school children play an important role during vaccination campaigns by bringing dogs to the vaccination centres in Namibia [21]. In turn, this observation would also explain why the grid cells/area that had a higher number of schools were associated with an increased vaccination coverage. Interestingly, adult, and male dogs were also associated with higher vaccination coverages, but the male dogs were removed from our model due to correlation issues. Our observation was that the male adult dogs were considered more valuable for the households/community compared to females and juvenile dogs. This observation is not surprising as previous studies have shown that adult male dogs are used for security and therefore given preferences when vaccination campaigns are underway [19,44,45]. Nevertheless, our study also demonstrated almost an equal proportion of females as well as juvenile dogs being presented for vaccination (Table 1). This suggested that the community in the NCA regions were aware of the importance of vaccinating their dogs against rabies irrespective of sex or age. This may be due to continuous awareness initiatives being provided to the community via radio broadcasts, TV programmes and through distribution and display of rabies posters since the implementation of rabies elimination program in 2016 [21]. Interestingly, the model showed that there was low vaccination coverage in more densely populated areas comparing to the less populated places. This observation could either be an effect of varying efficiencies of MDVs in different settings, the assumed human:dog ratio differing in the less populated places compared to densely populated places, or a combination of both. As explained above in the regression analysis methods, since the vaccination campaign was conducted by vaccination posts, some of the dog owners from outside the grid cells could have brought dogs to another nearby cell for vaccination. Thus, the number of dogs vaccinated per cell would not have necessarily corresponded with the number of vaccinated dogs living in the cell. Therefore, we have accounted for this by smoothing the data and conducting spatial regression. It is important to account for spatial autocorrelation and sampling variability when analyzing spatial data, particularly when there is sampling variability due to small populations or heterogeneity of individuals within areas when the areal units contain a small population [27].

Although our models identified a few critical predictors that influenced vaccination coverage, the inclusion of more variables in the model could provide valuable insight (e.g. the distance between the dog owners’ household and the vaccination centres, knowledge and awareness level of the dog owners with regards to rabies, education level of the dog owners, income level of the dog owners, dog management condition etc.,). We would plan to gather such data in future–i.e. detailed dog population studies in various socio-economic setting in order to obtain better quality baseline data on dog population density and turnover which would also allow us to measure the vaccination coverage more accurately [46]. In addition, post-vaccination surveys to assess the vaccination coverage and the validity of the gridded cell/area approach are also being planned for future implementation.

With 1.2 million people spread out over 263,376 km^2^ the dog rabies control program in the NCAs is logistically challenging, also considering the monitoring of the campaigns [21]. Initially, mobile phone technology using the EpiCollect platform [31,32] had been implemented to attempt to monitor MDV campaigns, but the wide-scale implementation failed due to lack of government supplied mobile phones. Thus, the GDL was the preferred choice because of its versatility, ease of handling and the constant technical support provided by GARC [24]. Despite overcoming many of the shortcomings associated with mobile phone technology, one limitation to the use of the GDL devices was the inability to upload data to the REB in the field if a laptop with an internet connection was not available. Since the memory capacity of the GDL is limited to 500 vaccination records, the vaccination team had to return to their local office to download the data and to clear the data from the device before preparing for the next day’s vaccination centers. This caveat was of less of a concern in this study as none of the vaccinators had managed to vaccinate more than 500 animals in a single day during the 2019 and 2020 MDV campaign. As such, the devices could be used without interruption. However, it may pose a challenge if vaccinator efficiency is higher as was observed during the pilot phase of Namibia’s vaccination campaign (580 dogs per day) [21] or in other countries including Uganda, Malawi and India where 596, 1760 and 4500 dogs were vaccinated per day respectively [16,31,33]. This only applies to one vaccinator or one team using a single GDL device–larger numbers of daily vaccinations are typically the work of multiple teams, each of which can use one or more dedicated GDL’s. Nevertheless to overcome a potential limitation posed by the GDL’s limited current internal memory, GARC has developed an offline version of the software that downloads the data to a laptop in a format that is ready for upload at a later time when an internet connection is available. In addition, GARC has developed a freely available mobile phone application that relies on a user-friendly interface that is similar to that of the GDL to collect vaccination data. In addition to relying on a similar interface, the mobile phone application allows far more data to be stored and uploaded to the REB whenever a stable internet connection is available.

Due to the government’s hesitation to use mobile phone, the GDL was the only viable option for the Namibian rabies elimination project. Initially, we procured only 20 GDL units for the vaccination campaign at a cost of approximately USD100 per unit. The cost of each device was, however, a once-off investment with the cost subsequently being distributed across each vaccinated animal. As such, the initial investment in the 20 GDL units resulted in an additional estimated cost of only 0,07 USD per vaccinated animal after the first two years of operation. While this cost is almost negligible, the additional cost per vaccinated animal will decrease even further as the devices are used in future campaigns. Moreover, only 20 additional GDL units will be required to cover the remaining regions–Kavango East, Kavango West, Kunene, Zambezi–ensuring that the vaccination data can be collected throughout the NCA (Fig 1A).

As is the case with using any technological device in harsh environments, some malfunctioning was encountered during the campaign. More specifically, one of the GDLs had malfunctioned during the campaign causing the data to corrupt. The loss of data was resolved by counting the number of spent vaccine doses at the vaccination stations, and manually entering the data on the REB. Despite the benefits and limitations associated with the use of newer technology, the continued future implementation of modern data digital data collection tools such as the GDL and sophisticated real-time data analysis will enable the Namibia government to take greater strides towards the elimination of dog-mediated rabies by 2030. The ability to easily and accurately track and trace each individual dose of vaccine, and in so doing each and every animal vaccinated, is a powerful capacity that should form part and parcel of all future vaccination campaigns. As studies such as our work in Namibia described here–as well as others across the world now demonstrate–the benefits of such data are overwhelming. In addition, the cost of vaccine/vaccination tracking is so negligible in comparison to the overall campaign cost, that it would be hard to rationalize any major future campaigns that do not use a digital vaccination tracking approach.

## Supporting information

S1 Appendix Explanation on OLS, spatial regression model and model diagnostics. (DOCX)

S1 Data Number of dogs vaccinated and estimated vaccination coverage at the 20x20 KM grid cells in the Northern Communal Area of Namibia, 2019–2020. (CSV)

S1 Table Results of Ordinary Least Square model, Spatial Lag Model and Spatial Error Model to assess the variables associated with a log-transformed grid level (20 x 20 km) vaccination coverage against rabies in dogs during 2020 mass dog vaccination campaign in NCA regions, Namibia. (DOCX)

S2 Table Results of Ordinary Least Square model, Spatial Lag Model and Spatial Error Model to assess the variables associated with a log-transformed grid level (20 x 20 km) vaccination coverage against rabies in dogs during 2019 mass dog vaccination campaign in NCA regions, Namibia. (DOCX)

S1 Fig Map displaying the locations of vaccination points during the vaccination campaign in 2019 (red dots) and in 2020 (black dots) in the four regions (Oshana, Oshikoto, Omusati and Ohangwena) as downloaded from Rabies Epidemiological Bulletin. The REB allows real time online visualization of vaccination points.
(TIF)

S2 Fig Map displaying the locations of schools in the four regions (Oshana, Oshikoto, Omusati and Ohangwena) of Northern Communal Area, Namibia (data updated as of 25/11/2015) and downloaded from the data world [47]. (TIF)

S3 Fig The bar graph and time distribution of animal vaccination during 2019 (A and B) and 2020 (C and D) vaccination campaigns in the four regions (Oshana, Oshikoto, Omusati and Ohangwena) of Northern Communal Area, Namibia. (TIF)
