# Supplementary material for: Application of the GARC Data Logger—a custom-developed data collection device—to capture and monitor mass dog vaccination campaigns in Namibia
Source: PLoS Negl Trop Dis. 2020 Dec 28;14(12):e0008948. doi: 10.1371/journal.pntd.0008948 (PMC7793283; doi:10.1371/journal.pntd.0008948)
Supplement: S1 Appendix — (DOCX) [file pntd.0008948.s001.docx]

**S1 Appendix.**

**Explanation on OLS, spatial regression model and model diagnostics**

The descriptive statistics was conducted for the outcome (vaccination coverage) and the predictors variables. The distribution of the grid cell level smoothed vaccination coverage rates was right-skewed, a log-transformation normalized this distribution (Shapiro-Wilk normality test 0.605, P=0.861 for 2020 data and 0.978 P=0.512 for 2019 data). The predictor variables were also log transformed to normalize the distribution.

We used GeoDA 0.9.5-i5(Beta) software to analyse the data [1]. First, a forward step wise classic ordinary linear regression (OLS) model was fit to the data using first order rook contiguity weight matrix and assessed the model [1]. The best-fitting ordinary least-squares model of log-transformed vaccination coverage rate included the number of schools present, estimated human population, and number of adult dog population for 2020 campaign performance while the estimated human population and number of adult dog population explained the 2019 vaccination coverage performance (S1 Table and S2 Table). We then examined the model residuals and were found to be normally distributed (Jarque-Bera test statistic 0.666, P=0.716) and heteroskedastic (Breusch-Pagan test statistic 4.554, P=0.207) for 2020 vaccination data and also for 2019 data (Jarque-Bera test statistic 3.893, P=0.143; Breusch-Pagan test statistic 5.124, P=0.077).

Next, the Moran’s I statistic was examined to identify residual autocorrelation in the best-fitting regression model [1]. A Moran’s I value near to +1 is indicative of clustering and a value near –1 is indicative of dispersion. A value close to zero indicates that the pattern of features is spatially random [2]. Presence of spatial autocorrelation in the residuals of regression model can result in model misspecification. If the residuals are autocorrelated, spatial regression models need to be built to take into account these spatial relationships. This can be achieved by running spatial lag and spatial error regression models in which these spatial regression models directly include spatial dependency within the regression analysis by either including a spatially lagged dependent variable, or including the spatial correlation within the model error term [1]. The best fit model to the data can then be assessed based on model diagnostic tests for spatial dependence [1]]. GeoDa provides a range of diagnostics to detect spatial dependence.

In our analysis, the OLS model residuals contained no significant autocorrelation for both 2020 (Moran’s I = 0.387, P=0.698) and 2019 data (Moran’s I = -0.137, P=0.890). However, the common diagnostics tests for spatial dependence - standard Lagrange multiplier test statistics for the spatial lag, robust Lagrange multiplier lag, and the robust Lagrange multiplier error were significant (P<00001) while the Lagrange Multiplier (error) is not significant for both 2020 and 2019 data, indicating the presence of spatial dependence, and suggesting that a spatial lag model specification via a maximum likelihood (ML) be estimated next [1]. The spatial dependence occurs when a value observed in one location depends on the values observed at neighboring locations. It also provides unbiased regression estimates using a Maximum Likelihood approach (ML Spatial Lag or Spatial Error models) [1].

After we identified the presence of spatial dependence, and to prevent model misspecification, we investigated both Spatial Lag and Spatial Error regression models with maximum likelihood approach while controlling for the spatial dependence. The best fit model was selected based on log likelihood, AIC and diagnostic tests for spatial dependence [1]. As explained above, the spatial relationships (spatial weights) between grid cell log-transformed smoothed vaccination coverage rates were defined by first order rook contiguity spatial weights. The results of both the Spatial Lag and Spatial Error models is presented in S1 Table and S2 Table.

The spatial lag term of the grid level vaccination coverage appeared as an additional indicator in the model. The coefficient parameter (Rho‘ρ’) reflects the spatial dependence inherent in our data, measuring the average influence on observations by their neighbouring observations. It has a positive effect and is highly significant for both 2020 and 2019 campaign coverage rate [1]. As a result, the general model fit improved, as indicated in the higher values of R-squared (pseudo-R^2^) and Log likelihood (S1 Table and S2 Table). The effects of other independent variables remain virtually the same. However, the significance of Breusch-Pagan test, the Likelihood Ratio test and the Moran’s I statistics of spatial lag dependence for 2019 data (S2 Table) and the significance of Likelihood Ratio test and the Moran’s statistics for 2020 data (S1 Table) suggests that although the introduction of spatial lag term improved the model fit, it did not make the spatial effects go away. This suggest that a further consideration is needed of alternative speciﬁcations, either including new explanatory variables or incorporating diﬀerent spatial weights, but we did not incorporate the new explanatory variables and also different spatial weights in our analysis [1].

Next, we built the spatial error model. A coefficient on the spatially correlated errors (LAMBDA, ‘λ’) was added as an additional indicator. It has a positive effect and was significant for 2020 coverage but not for 2019 coverage data. Like the lag model, the effects of other independent variables remain virtually the same. The Breusch-Pagan test, the Likelihood Ratio test and the Moran’s I statistics of spatial error dependence for both 2019 and 2020 vaccination coverage (S1 Table and S2 Table) were not significant suggesting that the introduction of spatial error term make the spatial effects go away, and also improved the model fit comparing to the OLS.

Comparing between the spatial lag and spatial error models, the spatial lag model better improved the model fit. Finally, a spatial lag model containing the number of schools, estimated human population, and the number of adult dogs fit the 2020 data (pseudo-R^2^: 0.517; log likelihood: 10.2638, Akaike info criterion: -10.5277) and 2019 data (pseudo-R^2^: 0.7533; log likelihood: 25.5953, Akaike info criterion: -43.1905) explaining the vaccination coverage performance in Namibia (S1 Table and S2 Table). In our investigation, there was an inverse relationship between the population density and vaccination coverage in both 2019 and 2020 data, indirectly suggesting that there would be a low coverage with increasing dog population if the existing human:dog ratio is considered.

In addition, an exploratory analysis was conducted using the model predicted values and residuals to assess the model fitness. The observed and predicted smoothed log-transformed grid level vaccination coverage rates were highly correlated (r:0.8995, P<0.0001) for 2019 data and moderately correlated for 2020 data (r:0.5651, P<0.0001) indicating the fit of the model.

**References**

1. Anselin L (2005) Exploring Spatial Data with GeoDa™: A Workbook. University of Illinois, Urbana Champaign. Available at: <http://www.csiss.org/clearinghouse/GeoDa/geodaworkbook.pdf>
2. Ward MP, Carpenter TE (2000) Techniques for analysis of disease clustering in space and in time in veterinary epidemiology. Prev Vet Med 45: 257-284. 10.1016/s0167-5877(00)00133-1
