# Supplementary material for: Application of the GARC Data Logger—a custom-developed data collection device—to capture and monitor mass dog vaccination campaigns in Namibia
Source: PLoS Negl Trop Dis. 2020 Dec 28;14(12):e0008948. doi: 10.1371/journal.pntd.0008948 (PMC7793283; doi:10.1371/journal.pntd.0008948)
Supplement: S1 Table — (DOCX) [file pntd.0008948.s003.docx]

**S1 Table:** Results of Ordinary Least Square model, Spatial Lag Model and Spatial Error Model to assess the variables associated with a log-transformed grid level (20 x 20 km) vaccination coverage against rabies in dogs during 2020 mass dog vaccination campaign in NCA regions, Namibia.

|  | **Ordinary Least Square model (Aspatial)** | | | | **Spatial Lag Model** | | | | **Spatial Error Model** | | | |
| --- | --- | --- | --- | --- | --- | --- | --- | --- | --- | --- | --- | --- |
| ***Variable*** | ***Coefficient*** | ***SE*** | ***Z-value*** | ***P-value*** | ***Coefficient*** | ***SE*** | ***Z-value*** | ***P-value*** | ***Coefficient*** | ***SE*** | ***Z-value*** | ***P-value*** |
| Spatial Lag (Rho) and Spatial Error (Lamda) |  |  |  |  | 0.39303 | 0.11049 | 3.55700 | 0.00037 | 0.32240 | 0.12643 | 2.54992 | 0.01077 |
| Constant | 0.97235 | 0.45651 | 2.12995 | 0.43594 | 0.75294 | 0.39858 | 1.88905 | 0.05888 | 0.59906 | 0.48609 | 1.23241 | 0.21779 |
| Number of schools | 0.43594 | 0.17137 | 2.54386 | 0.01355 | 0.31687 | 0.15336 | 2.06613 | 0.03881 | 0.36022 | 0.17962 | 2.00548 | 0.04491 |
| Human population | -0.68599 | 0.15123 | -4.53582 | 0.00003 | -0.47104 | 0.13865 | -3.39733 | 0.00068 | -0.51859 | 0.16078 | -3.22530 | 0.00125 |
| Adult dog population | 0.24244 | 0.04834 | 5.01556 | 0.000005 | 0.15184 | 0.04261 | 3.56349 | 0.00037 | 0.11940 | 0.04627 | 2.58016 | 0.00987 |
| Number of observations | 64 |  |  |  | 64 |  |  |  | 64 |  |  |  |
| Log likelihood | 5.43006 |  |  |  | 10.2638 |  |  |  | 5.53148 |  |  |  |
| Akaike information criterion (AIC) | -2.86012 |  |  |  | -10.5277 |  |  |  | -3.06297 |  |  |  |
| R square (pseudo-R^2^) | 0.37413 |  |  |  | 0.517521 |  |  |  | 0.429019 |  |  |  |
| Jarque-Bera Test |  |  |  | 0.71599 |  |  |  |  |  |  |  |  |
| Breusch-Pagan Test |  |  |  | 0.20682 |  |  |  | 0.28155 |  |  |  | 0.34431 |
| Likelihood Ratio Test |  |  |  |  |  |  |  | 0.00187 |  |  |  | 0.65243 |
| Moran's I test |  |  |  | 0.69728 |  |  |  | 0.007 |  |  |  | 0.244 |

**NOTE:**

Jarque-Bera is a test for normality and if the p value is not significant (>0.05), then the distribution is said to be normal.

Breusch-Pagan Test is a test to detect heteroskedasticity, i.e., a non-constant error variance. If the p-value is not significant, it is considered as heteroskedastic.

Likelihood Ratio Test is a test for spatial dependence and significant p-value indicate presence of spatial dependence in the model.

Moran's I statistic is a test for residual autocorrelation and significant p-value indicate model residual autocorrelation.

R square: The value listed in the spatial lag and error output is not a real R^2^, but a so-called pseudo-R2, which is not directly comparable with the measure given for OLS results. Greater the R^2^ value, better the model fit. Adjusted R^2^ value increases by adding additional explanatory variables.

Higher the Log Likelihood value and lower the AIC value, better the model fit.
