# Supplementary material for: Application of the GARC Data Logger—a custom-developed data collection device—to capture and monitor mass dog vaccination campaigns in Namibia
Source: PLoS Negl Trop Dis. 2020 Dec 28;14(12):e0008948. doi: 10.1371/journal.pntd.0008948 (PMC7793283; doi:10.1371/journal.pntd.0008948)
Supplement: S2 Table — (DOCX) [file pntd.0008948.s004.docx]

**S2 Table:** Results of Ordinary Least Square model, Spatial Lag Model and Spatial Error Model to assess the variables associated with a log-transformed grid level (20 x 20 km) vaccination coverage against rabies in dogs during 2019 mass dog vaccination campaign in NCA regions, Namibia

|  | **Ordinary Least Square Model (Aspatial)** | | | | **Spatial Lag Model** | | | | **Spatial Error Model** | | | |
| --- | --- | --- | --- | --- | --- | --- | --- | --- | --- | --- | --- | --- |
| ***Variable*** | ***Coefficient*** | ***SE*** | ***Z-value*** | ***P-value*** | ***Coefficient*** | ***SE*** | ***Z-value*** | ***P-value*** | ***Coefficient*** | ***SE*** | ***Z-value*** | ***P-value*** |
| Spatial error (Lamda) |  |  |  |  | 0.53777 | 0.10826 | 4.96702 | 0.00007 | -0.20392 | 0.16293 | -1.25154 | 0.21073 |
| Constant | 0.54774 | 0.19343 | 2.83172 | 0.00684 | 0.25664 | 0.15208 | 1.68758 | 0.09149 | 0.67137 | 0.16408 | 4.09164 | 0.00000 |
| Human population | -0.52480 | 0.06156 | -8.52485 | 0.00000 | -0.26109 | 0.05926 | -4.40546 | 0.00001 | -0.58365 | 0.05209 | -11.2029 | 0.00000 |
| Adult dog population | 0.384815 | 0.06132 | 6.27591 | 0.00000 | 0.21522 | 0.05176 | 4.15735 | 0.00003 | 0.44085 | 0.05545 | 7.94954 | 0.00000 |
| Number of observations | 49 |  |  |  | 49 |  |  |  | 49 |  |  |  |
| Log likelihood | 17.8852 |  |  |  | 25.5953 |  |  |  | 18.21165 |  |  |  |
| Akaike info criterion (AIC) | -29.7703 |  |  |  | -43.1905 |  |  |  | -30.4233 |  |  |  |
| R square (pseudo-R^2^) | 0.6056 |  |  |  | 0.7533 |  |  |  | 0.6324 |  |  |  |
| Jarque-Bera test |  |  |  | 0.142706 |  |  |  |  |  |  |  |  |
| Breusch-Pagan test |  |  |  | 0.077129 |  |  |  | 0.02471 |  |  |  | 0.06642 |
| Likelihood Ratio test |  |  |  |  |  |  |  | 0.00008 |  |  |  | 0.41905 |
| Moran's I |  |  |  | 0.890403 |  |  |  | 0.001 |  |  |  | 0.267 |

**NOTE:**

Jarque-Bera is a test for normality and if the p value is not significant (>0.05), then the distribution is said to be normal.

Breusch-Pagan Test is a test to detect heteroskedasticity, i.e., a non-constant error variance. If the p-value is not significant, it is considered as heteroskedastic.

Likelihood Ratio Test is a test for spatial dependence and significant p-value indicate presence of spatial dependence in the model.

Moran's I statistic is a test for residual autocorrelation and significant p-value indicate model residual autocorrelation.

R square: The value listed in the spatial lag and error output is not a real R^2^, but a so-called pseudo-R2, which is not directly comparable with the measure given for OLS results. Greater the R^2^ value, better the model fit. Adjusted R^2^ value increases by adding additional explanatory variables.

Higher the Log Likelihood value and lower the AIC value, better the model fit.
