# Supplementary figures and images for: Application of the GARC Data Logger—a custom-developed data collection device—to capture and monitor mass dog vaccination campaigns in Namibia
Source: PLoS Negl Trop Dis. 2020 Dec 28;14(12):e0008948. doi: 10.1371/journal.pntd.0008948 (PMC7793283; doi:10.1371/journal.pntd.0008948)

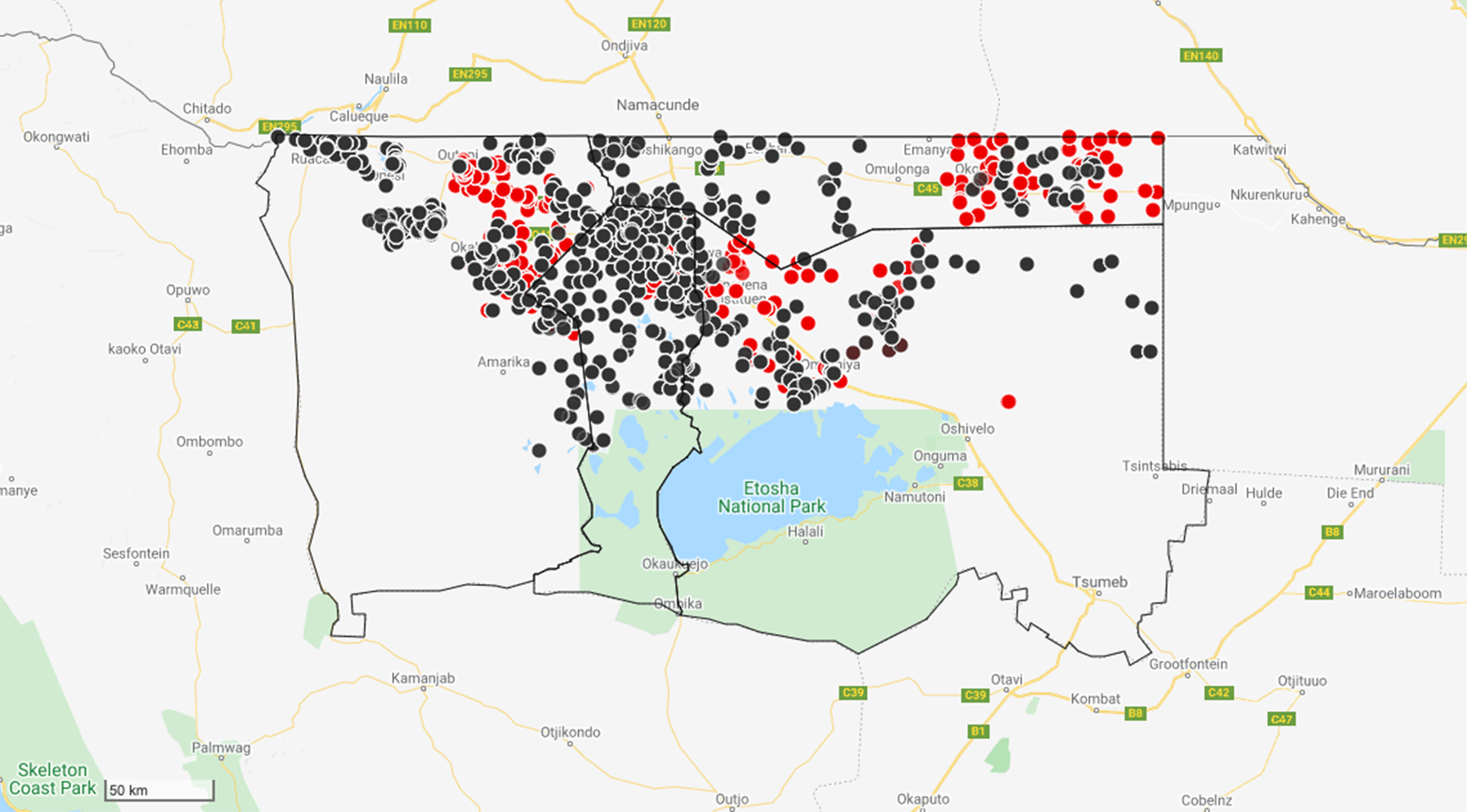

Supplement: S1 Fig — The REB allows real time online visualization of vaccination points. (TIF) [file pntd.0008948.s005.tif]

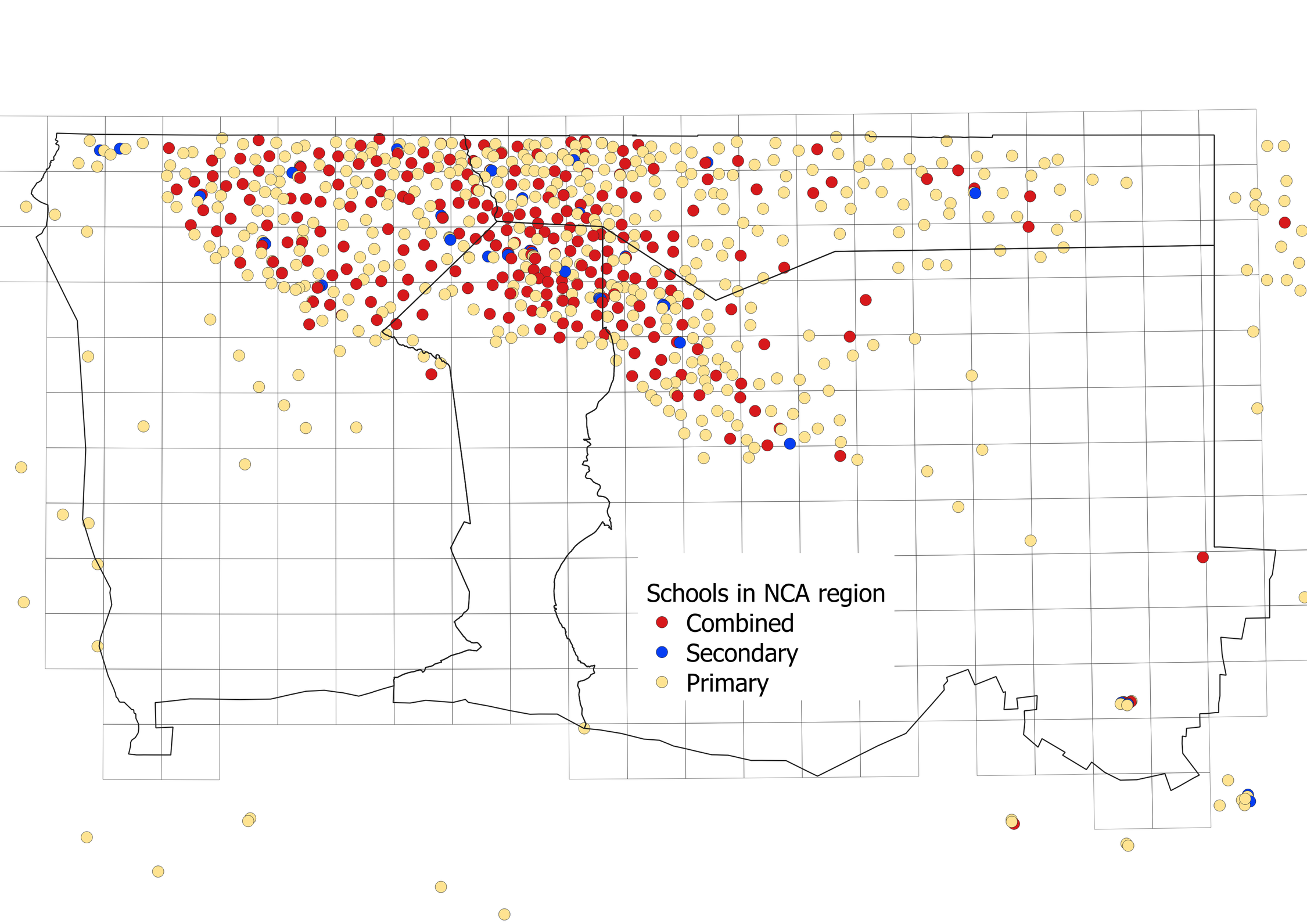

Supplement: S2 Fig — (TIF) [file pntd.0008948.s006.tif]

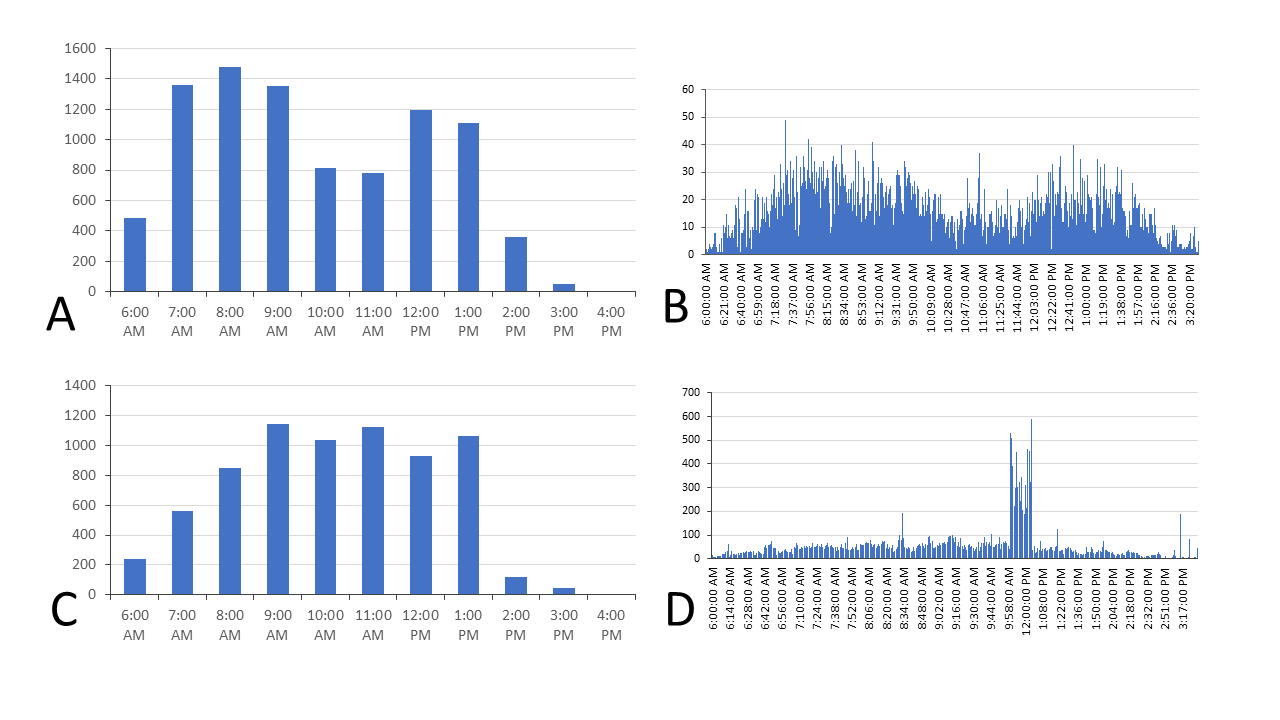

Supplement: S3 Fig — (TIF) [file pntd.0008948.s007.tif]
